# Depression induces poor prognosis associates with the down-regulation brain derived neurotrophic factor of serum in advanced small cell lung cancer

**DOI:** 10.18632/oncotarget.13291

**Published:** 2016-11-11

**Authors:** Yufeng Wu, Ruirui Si, Sen Yang, Suhua Xia, Zelai He, Lili Wang, Zhen He, Qiming Wang, Hong Tang

**Affiliations:** ^1^ Department of Internal Medicine, Affiliated cancer hospital of Zhengzhou University, Henan Cancer Hospital, Zhengzhou, Henan, 450008, P. R. China; ^2^ Department of Health Center, Henan Airport Group Co., Ltd., Henan, 450000, P. R. China; ^3^ Department of Oncology, The First Affiliated Hospital of Soochow University, Suzhou, Jiangsu, 215006, P. R. China; ^4^ Department of Oncology, The 2nd Affiliated Hospital and Yuying Children's Hospital of Wenzhou Medical University, Wenzhou, Zhejiang, 325000, PR China

**Keywords:** depression, chemotherapy, small cell lung cancer, brain derived neurotrophic factor, ABCG2

## Abstract

Patients with lung cancer often experience a state of depression, and these conditions may severely affect their quality of life (QoL) and prescription compliance. The current study was conducted to delineate the complex links between depression and the prognosis of patients with small cell lung cancer (SCLC) and the underlying mechanism was also explored.

186 patients who received platinum-based chemotherapy for newly diagnosed stage III or stage IV SCLC were enrolled. The Self-Rating Depression Scale (SDS) questionnaire was completed the day before the start of chemotherapy to assess the depression status of the patients. Patients with stage IV SCLC or lower BMI have higher depression scores. In terms of the adverse effects of chemotherapy, depression severely decreases patient tolerance to chemotherapy and their QoL score (R^2^ = 0.2385) and is also associated with severe vomiting (*P* < 0.001), leukopenia (R^2^ = 0.2332), and prolonged hospital stay (R^2^ = 0.1961). More importantly, severe depression reduces the PFS (R^2^ = 0.1943) and OS (*P* < 0.01) of the patients. We found that patients with severe depression displayed a downregulated level of serum BDNF and that the level of serum BDNF was highly correlated with the OS of the patients (R^2^ = 0.2292). Using the MTT cell viability assay *in vitro*, we observed that cotreatment with BDNF clearly enhanced the chemosensitivity of NCI-H69 tumor cells to Cisplatin and induced the downregulation of ABCG2.

Based on this evidence, it appears that a relationship does exist between depression and prognosis in SCLC and that the mechanism by which depression affects prognosis is achieved via the downregulation of BDNF expression.

## INTRODUCTION

Depression is a clinical mental disorder that can be both serious and life-threatening especially in patients with cancer. Depressive disorder is estimated to occur in 10%–25% of patients with cancer, which is greater than twice the rate in the general population [1]. Many emerging treatments have increased the survival of patients with cancer, but the quality of life (QoL) of those patients has not significantly increased [2, 3]. The effects of chemotherapy on SCLC patients are poor, and such treatments largely lead to financial difficulties and a lower quality of life. Specifically, patients who receive continuous periodic chemotherapy often experience long-term depression as a result of physical impairment and financial expenses. Obviously, depression and its influence on such patients deserve the greatest attention.

The psychological burden in patients with malignancies has gained increasing attention in the field of psycho-oncology. One of the most intriguing applications of the Self-Rating Depression Scale (SDS) scores is their use as prognostic factors. Buccheri found that patients with depression had lower rates of cancer survival than non-depressed patients [4]. In an early Scandinavian study, psychosocial wellbeing and general symptoms were found that significantly predicted the survival of patients with advanced non-small cell lung cancer [5]. However, the relationship between depression and prognosis required further investigation.

In recent years, diverse studies that have reported the assessment of QoL as a prognostic factor in cancer patients have been published. Ruckdesche analyzed the charts of 178 patients from four phase III trials, and confirmed the prognostic importance of QoL [6]. More recently, using a new instrument of assessment, Buccheri confirmed the supposed relationship between QoL and prognosis [7]. Most QoL dimensions are influenced by the psychological status of patients, which is seriously impacted by depression. The prognostic value of SDS scores might be a simple reflection of the severity of the illness, and because of the different psychological states, patients may have different prognoses based on the treatment they are given. It is therefore necessary to focus our research on the ways in which depression affects the prognosis of patients who receive chemotherapy.

Inoperable SCLC is typically treated with 6 cycles of platinum-based chemotherapy [8], but up to 75% of SCLC patients will relapse within 6 months due to the development of chemotherapy-resistant disease. Failure of effective chemotherapeutic treatment and the poor outcome of patients who undergo chemotherapy are partly due to the multidrug resistance of lung cancer cells. Depression is mentioned above as a prognostic factor in cancer patients, and preliminary studies have confirmed that benign psychological conditions can increase the chemosensitivity of tumors. Therefore, we hypothesize that depression downregulates tumor chemosensitivity and shortens the PFS and OS of patients with SCLC.

Recently, studies have suggested that brain derived neurotrophic factor (BDNF) plays an important role in the development of depression. As early as 1995, Duman's group found that anti-depressants and related medications increased the protein levels of BDNF and its receptor TrkB in rat brains [9]. A clinical study in 2004 showed that serum BDNF levels were negatively correlated with depression-related personality traits [10]. Afterwards, serum and plasma BDNF levels were found to be decreased in patients with depressive disorder [11]. Wang J indicated that Geniposide can alleviate depression-like behavior, which may partially be ascribed to the upregulation of BDNF expression in the brain [12]. Our previous clinical study showed that BDNF in the brain acts as an effector of transcription of immediate-early genes that are expressed as a response to eustress, which is thought to enhance the chemosensitivity of tumor cells [13, 14]. Studies that focus on depression and BDNF may provide insights that might facilitate the improvement of interventions for SCLC.

To better understand the effects of depression on SCLC and its mechanism in patients with SCLC, the present study was designed to address this issue through an analysis of the SDS scores of patients before chemotherapy, and through an investigation of the correlation between prognosis and depressive states. Because the determination of prognosis is complex and involves several components, we also investigated elements such as adverse effects of chemotherapy, QoL, PFS and OS, which may be affected by depression. Additionally, we evaluated the correlation between high levels of BDNF and chemosensitivity to platinum-based drugs *in vitro*. Then, we investigated the mRNA expression of genes, including ABCG2, that have been widely reported as chemoresistance-related genes in tumors. Once target genes were discovered, they were verified by protein analysis. Finally, we were able to demonstrate that ABCG2 was downregulated by BDNF.

## RESULTS

### Patient characteristics that affect depression

As shown in Table 1, the median age of the 186 patients with advanced SCLC who were included in the study was 66 years (range, 56–77 years), and approximately 80.1% of them were male. Patients with an SDS score > 50 are considered to have depression, and our results showed that the age and gender of the patients significantly affected the rates of depression. The average age of the patients with depression was significantly higher than the average age of all patients combined (69 ± 7.7 vs. 66.2 ± 6.3, *P* = 0.013). In terms of incidence, patients over the age of 65 had a significantly higher rate of depression [65/89 (73.03%) vs. 59/97 (60.82%), *P* = 0.0019]. Marital status and disease stage also affected the incidence of depression, as unmarried and stage IV patients had a much higher incidence of depression (unmarried 25/29 (86.20%) vs. married 99/157 (63.06), *P* = 0.015; stage IIIb 34/68 (50.00) vs. stage IV 90/116 (77.59), *P* < 0.001). Interestingly, patients with a high body fat percentage have a low incidence of depression. In our study, the depression rate was 38/45 (84.44%) in patients with an initial BMI < 18.5, while the depression rate was 29/52 (55.77%) (*P* < 0.001) in patients with a BMI of 18.5-24.99 57/89 (64.04%) and a BMI of >24.99.

**Table 1:** Demographic characteristics of patients and one way ANOVAs (n: Total participants; N: Participants with depression)

Patient Variables (*n* = 84)	*n* = 186 n1 (n1/n × 100)%	Depression	
		*N* = 124 n2(n2/n1 × 100)%	*p*-value
Age, years	66.2 ± 6.3	69 ± 7.7	0.013Δ
> 65	89 (47.85)	68 (73.03)	0.0019**
< 65	97 (52.15)	53 (60.82)	
Gender			
Male, n	37 (19.89)	24 (64.86)	0.795
Female, n	149 (80.10)	100 (67.11)	
Marital status			
Unmarried	29 (15.59)	25 (86.20)	0.015*
Married	157 (84.41)	99 (63.06)	
Family history of malignancy, n (%)			
Yes	39 (20.97)	27 (69.23)	0.7024
No	147 (79.03)	97 (65.99)	
Stage of disease			
IIIb	68 (36.56)	34 (50.00)	0.000***
IV	118 (63.44)	90 (76.27)	
WBC count (n/m^3^, initial)	5638±1723	5106±2019	> 0.05
BMI (initial)			
< 18.5	45 (24.19)	38 (84.44)	0.000***
18.5–24.99	89 (47.85)	57 (64.04)	
> 24.99	52 (27.96)	29 (55.77)	
Serum haemoglobin (g/dL, initial)	123.8 ± 1.6	128 ± 2.3	> 0.05
Serum LDH level (mg/dL, initial)	457 ± 452	371 ± 205	> 0.05
Serum CEA level (ng/mL, initial)	25.5 ± 8.6	29.3 ± 9.2	> 0.05

Δ Results are presented as Mean ± SD. Numbers in parentheses represent the numbers of patients accessible for analysis. *P < 0.05, **P < 0.01, ***P < 0.001

In contrast, we found no differences in terms of gender, WBC count, family history of malignancy, serum CEA level or serum LDH level in patients with different levels of depression (*P* > 0.05). The median serum hemoglobin, LDH and CEA values at baseline were 123.8 ± 1.6 g/dL, 457 ± 452 mg/dL, and 25.5 ± 8.6 ng/mL, respectively.

### Adverse effects on chemotherapy and quality of life based on depression status

**Figure 1:**
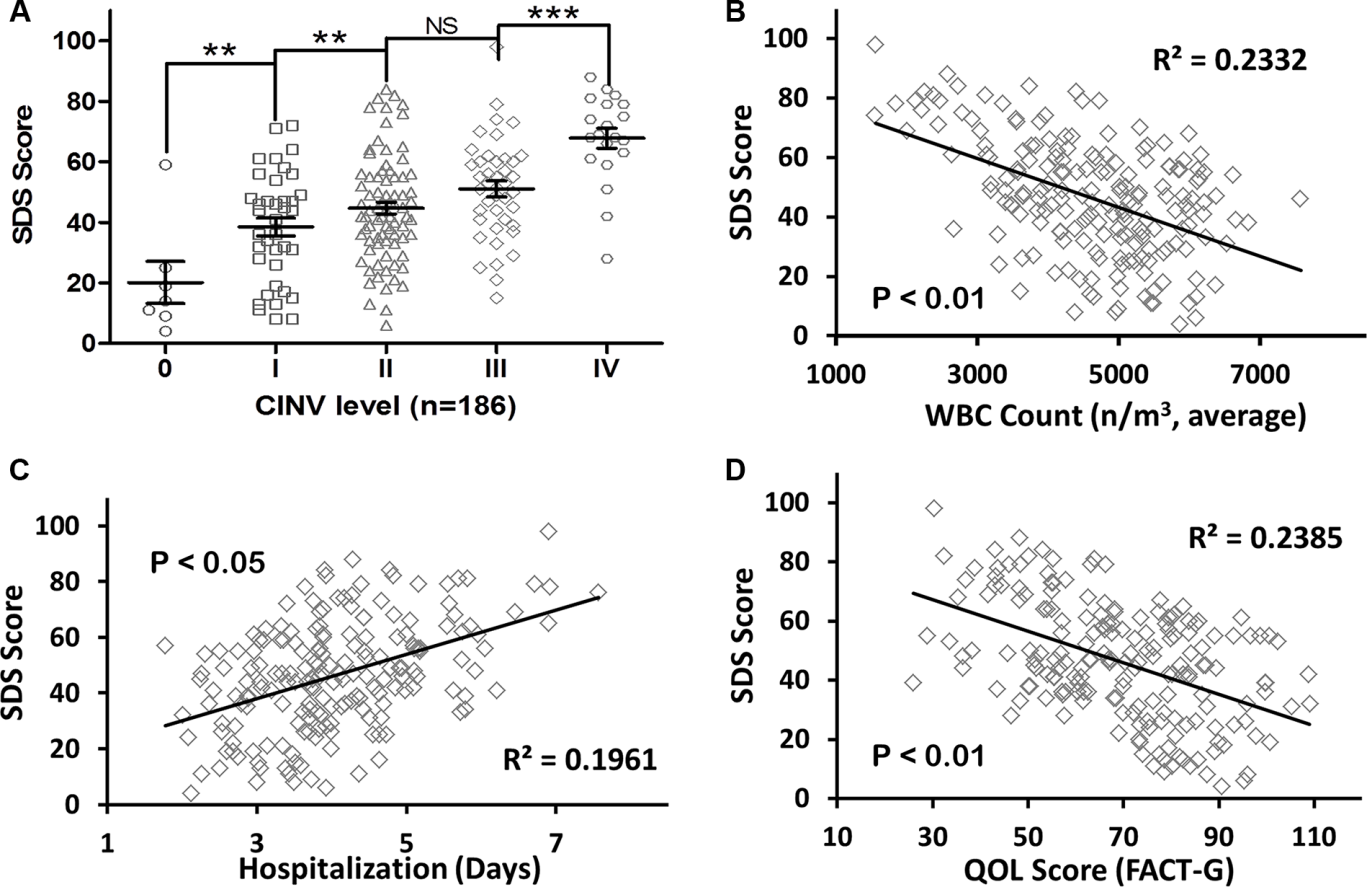
Depression status increased the adverse effects of chemotherapy and decreased the quality of life (**A**) SDS score increased significantly with a rise in the level of CINV. (**B**) an inverse correlation between depression and leukopenia was observed, which indicates that depression significantly reduced the incidence of leukopenia in patients with SCLC. (**C**) depression significantly prolonged the hospitalization stay of patients. (**D**) depression significantly reduced the quality of life of patients.

We observed adverse effects in patients who underwent chemotherapy, and then we performed a comparison and correlation analysis using the incidence and severity of depression in patients (SDS scores). Most patients (179/186, 96%) experienced varying degrees of chemotherapy-induced nausea and vomiting (CINV). As shown in Figure 1A, a comparison of the different groups showed a significant difference between the level of CINV and the SDS score of the patients. For example, the SDS score significantly increased as the level of CINV rose (Grade IV 67.80 ± 3.280 vs. Grade III 51.05 ± 2.597, *P* = 0.0003; Grade I 38.50 ± 2.925 vs. Grade 0 20.14 ± 6.971, *P* = 0.0177).

Our study showed that depression greatly prolongs chemotherapy recovery and reduces the quality of life of patients. Four WBC count test results were averaged for the analysis. A significant inverse correlation between depression and leukopenia was observed (R^2^ = 0.2332, *P* < 0.01), which suggests that depression reduced the incidence of leukopenia in the SCLC patients in this study (Figure 1B). Moreover, depression significantly prolonged the hospitalization stay of patients (R^2^ = 0.1961, *P* < 0.05) and significantly reduced the quality of life of patients (R^2^ = 0.2385, *P* < 0.01).

### Survival outcomes based on depression status

**Figure 2:**
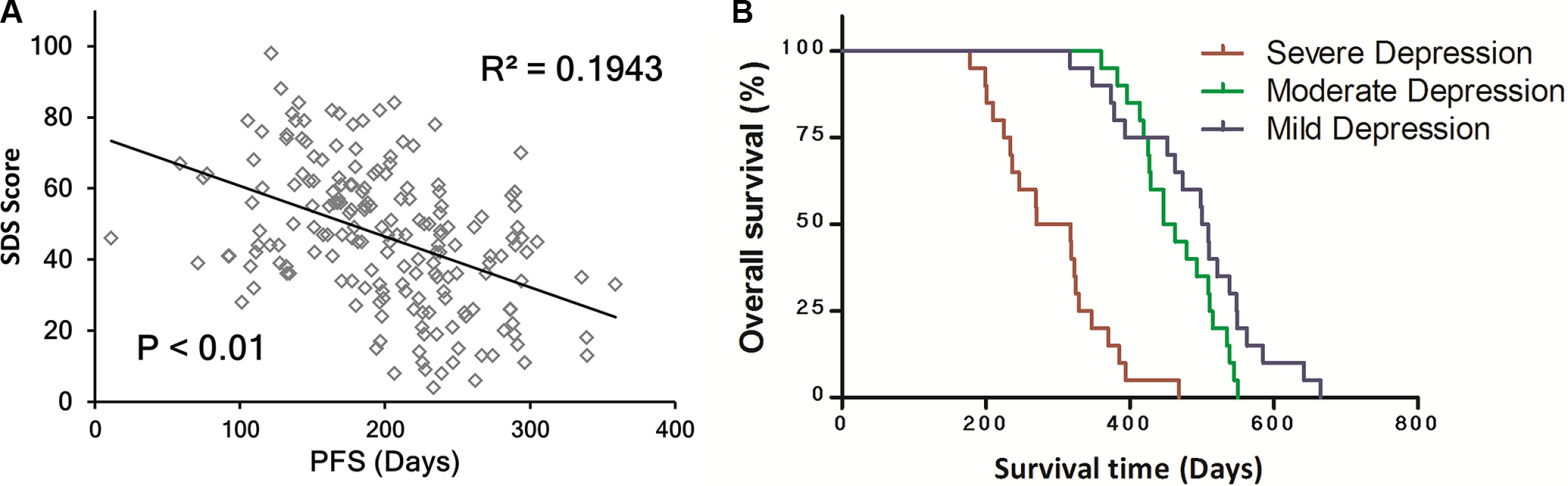
Depression reduced progression-free survival time and overall survival outcomes (**A**) A correlation analysis revealed a significant negative correlation between depression and progression-free survival. (**B**) patients were divided into three group according to SDS scores, and patients in the mild depression group had a longer survival time than patients in the severe and moderate depression groups (*P* < 0.01).

A correlation analysis was used to uncover the relationship between depression and progression-free survival. The analysis showed a significant negative correlation between these two factors (R^2^ = 0.1943, *P* < 0.01), which indicates that more severe depression induced a significantly shorter PFS (Figure 2A).

In order to determine the influence of depression on overall survival, three groups of patients with different levels of depression were enrolled according to their SDS scores, as follows: the mild depression group (SDS score < 40, *n* = 20), the moderate depression group (SDS score between 45 and 65, *n* = 20), and the severe depression group (SDS score > 75, *n* = 20). After a Kaplan-Meier analysis and log-rank test were performed, as is shown in Figure 2B, it was revealed that patients in the mild depression group had a longer survival time than those in the severe and moderate depression groups (*P* < 0.01). However, no significant difference was observed between the moderate group and the mild group (*P* > 0.05). This means that depression significantly reduced the survival time of these patients.

### Depression mediated the BDNF level of patients

**Figure 3:**
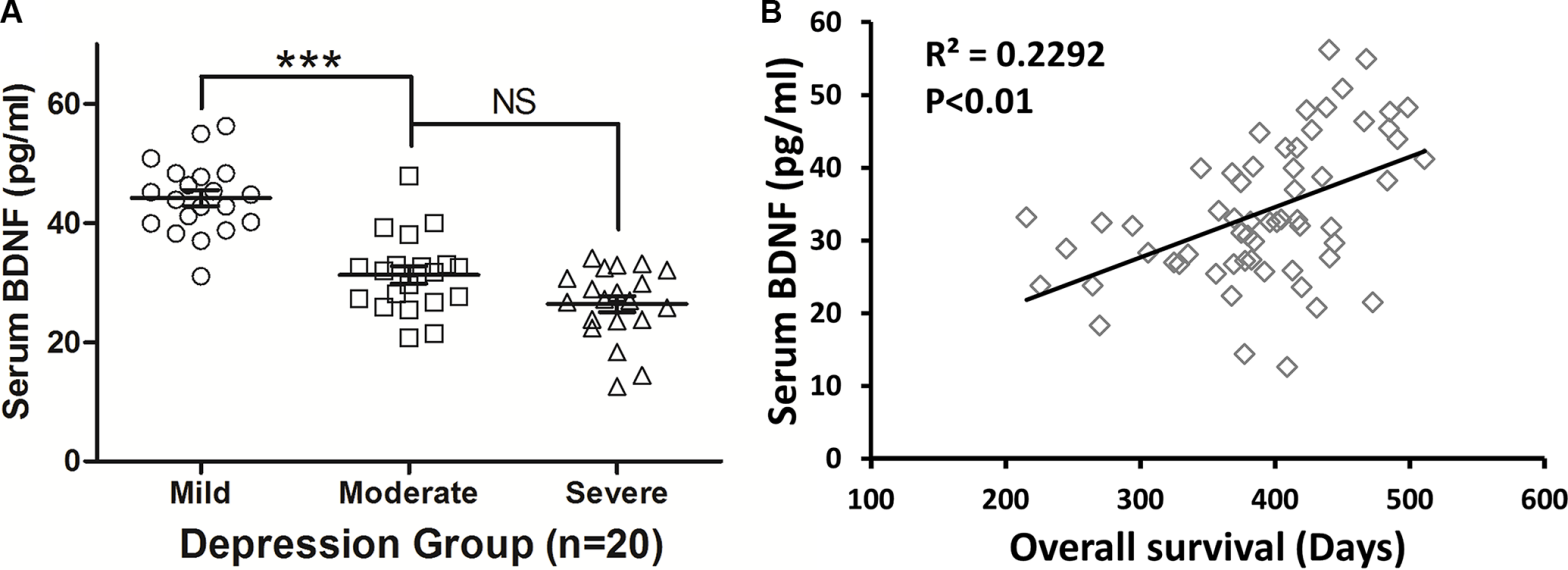
Depression mediated the BDNF level in patients (**A**) Patients with mild depression had a significantly higher level of serum BDNF than those with moderate and severe depression. However, the level of serum BDNF between the moderate depression group was not significantly different from that of the severe depression group. (**B**) BDNF demonstrated a significant positive correlation with OS, and a higher serum BDNF level prolonged the OS of patients.

BDNF is considered an effector of the transcription of immediate-early genes, which are expressed in response to depression. In order to further confirm the effects of depression on the central nervous system (CNS) in patients with SCLC, we measured the serum BDNF levels and found that increased depression was associated with significant decreases in BDNF levels. As shown in Figure 3A, patients with mild depression had a significantly higher level of serum BDNF (45.283 ± 4.213 pg/ml) than those with moderate (33.178 ± 4.289 pg/ml, *P* < 0.001) or severe depression (28.190 ± 3.143 pg/ml, *P* < 0.001). However, the level of serum BDNF in the moderate depression group was not significantly different from the severe depression group (*P* = 0.673, Figure 3A), which is consistent with the OS of the patients (Figure 2B). Furthermore, we analyzed the relationship between the serum BDNF level and OS. A significant positive correlation was found between serum BDNF and OS (R^2^ = 0.2292, *P* = 0.0046), which suggests that a higher serum BDNF level could prolong the OS of these patients (Figure 3B). This means that depression significantly reduced the level of serum BDNF and that it indirectly affected the prognosis of the patients via BDNF regulation.

### BDNF increased the chemosensitivity of NCI-H69 cells

**Figure 4:**
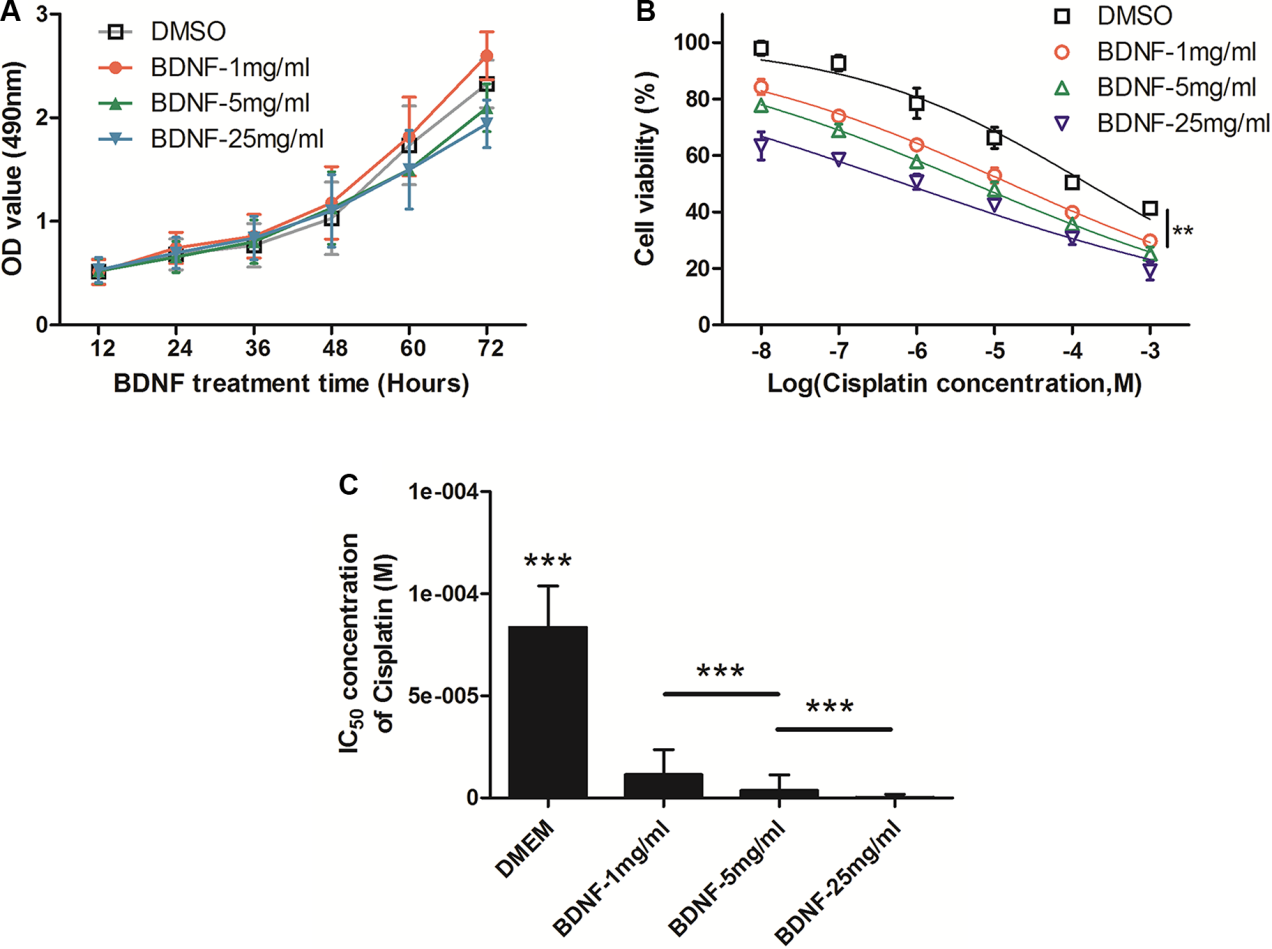
BDNF increased the chemosensitivity of NCI-H69 cells (**A**) Water-soluble BDNF at 3 concentrations (1 mg/ml, 5 mg/ml, 25 mg/ml) had no significant effect on cell proliferation. NCI-H69 cells were cultured in medium with different concentrations of BDNF (1 mg/ml, 5 mg/ml or 25 mg/ml) and were incubated for 24 hours; they were then tested for chemosensitivity to Cisplatin by MTT assay. (**B**) Chemosensitivity was significantly increased in the BDNF group after exposure to Cisplatin and demonstrated a dose-effect relationship. (**C**) The IC_50_ of NCI-H69 cells cultured with BDNF was clearly decreased compared with that in control cells. (^*^*P < 0.01, ^**^*P < 0.001).

In order to verify whether BDNF affects cell proliferation and chemosensitivity in SCLC, NCI-H69 cells were selected for further study. Water-soluble BDNF was added to the cell culture medium in 3 concentrations (1 mg/ml, 5 mg/ml or 25 mg/ml). Then, the proliferation of NCI-H69 cells was analyzed by MTT every 12 hours; cells cultured in normal medium served as controls. The cell proliferation curves show that BDNF had no significant effect on cell proliferation (Figure 4A).

To test chemosensitivity, suspension NCI-H69 cells were cultured in medium with different concentrations of BDNF (1 mg/ml, 5 mg/ml or 25 mg/ml) and were incubated for 24 hours. Then, the cells were treated with increasing concentrations of Cisplatin (10^−8^, 10^−7^, 10^−6^, 10^−5^, and 10^−4^ M). As shown in Figure 4B, cell chemosensitivity was significantly increased in the BDNF-1 mg/ml group after exposure to Cisplatin, and the cells that were treated with higher concentrations of BDNF exhibited higher sensitivities. The half-maximal inhibitory concentration (IC_50_) of Cisplatin was then analyzed in these tumor cells via the application of a previously described method. As expected, the IC_50_ values of NCI-H69 cells cultured with BDNF were obviously decreased, while the IC_50_ of DMSO was 216.82 ± 17.25 μmol/L. The IC_50_ value of BDNF-1 mg/ml was 23.75 ± 1.95 μmol/L (vs. DMSO, *P* < 0.001), the IC_50_ value of BDNF-5 mg/ml was 7.19 ± 1.43 μmol/L (vs. BDNF-1 mg/ ml, *P* < 0.001), and the IC_50_ of BDNF-25 mg/ml was 0.82 ± 0.13 μmol/L (vs. BDNF-5 mg/ml, *P* < 0.001), which are shown in Figure 4C and Table 2. Taken together, these results provide evidence that BDNF led to a considerable increase in chemosensitivity of NCI-H69 cells, which was in turn regulated by depression status.

**Table 2:** NCI-H69 cells cultured in high BDNF significantly decreased the resistance to Cisplatin

	IC_50_ to Cisplatin (μmol/L)	*P*-value
DMSO	216.82 ± 17.25	< 0.001***
BDNF-1 mg/ml	23.75 ±1.95	< 0.001***
BDNF-5 mg/ml	7.19 ± 1.43	< 0.001***
BDNF-25 mg/ml	0.82 ± 0.13	< 0.001***

### BDNF regulated chemosensitivity via ABCG2

In order to investigate the mechanism of BDNF influence on chemosensitivity, we used real-time PCR to screen mRNAs of genes that have been widely reported to be involved in the development of drug-resistance. The following 9 candidate genes were chosen for further validation: GST-p1, GSTm1, ABCG2, PPARa, DPYD, ABCB1, ABCC1, ABCC2, and ABCC9. We examined the mRNA expression of these 9 candidates in NCI-H69 cells that were cultured with BDNF (25 mg/ml) for 12 hours; cells cultured with DMSO served as controls.

**Figure 5:**
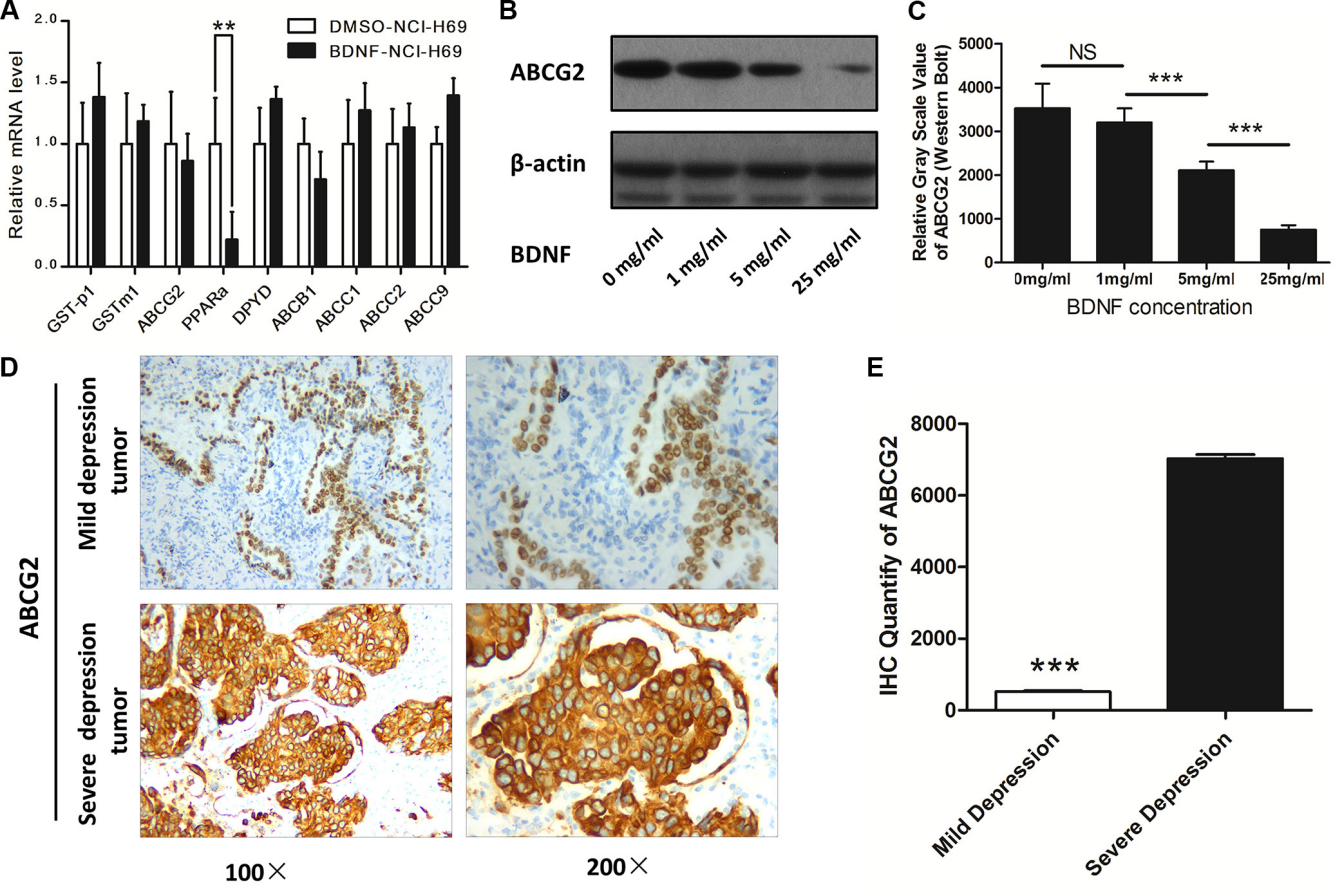
ABCG2 was downregulated by BDNF and depression (**A**) The mRNA expression of ABCG2 was downregulated in NCI-H69 cells co-cultured with BDNF (25 mg/ml); then, NCI-H69 cells were exposed to different concentrations of BDNF in culture (1 mg/ml, 5 mg/ml or 25 mg/ml). (**B**) Western blot analysis revealed that cells in the BDNF culture group showed a marked decrease in ABCG2 expression. (**C**) The relative ABCG2 expression in cells cultured with BDNF was quantified by Western blot and was found to be significantly higher than that in the controls. (**D**) Immunohistochemical analysis revealed that the tumors of patients in the severe depression group demonstrated strong expression of ABCG2. (**E**) The % positive area for ABCG2 staining in tumors from patients with severe depression was 15.6-fold higher than in patients with mild depression.

As the result in Figure 5A shows, out of the eight candidate genes in BDNF-cultured (25 mg/ml) cells, the mRNA level of ABCG2 was significantly lower than that in control cells (1.000 ± 0.251 vs. 4.256 ± 0.325, *P* < 0.001). In addition, the mRNA expression of other candidate genes showed no significant difference (*P* > 0.05).

In order to validate the qPCR results, we tested the expression of ABCG2 protein in NCI-H69 cells cultured with different concentrations of BDNF (1 mg/ml, 5 mg/ml, and 25 mg/ml). Western blot analysis revealed that cells cultured with BDNF showed a marked decrease in ABCG2 protein (Figure 5B), and as the concentration of BDNF increased, the level of ABCG2 protein decreased. As the grayscale analysis shows, ABCG2 protein expression in the control group was 4.56-fold higher than that in the BDNF-25 mg/ml group (Figure 5C).

We next explored the expression of ABCG2 using immunohistochemistry in tumors from patients with mild depression and from those with severe depression (*n* = 10). The results were in agreement with the previous data, and demonstrated that the tumors of patients with severe depression demonstrated stronger expression of ABCG2 (Figure 5D). ImageJ analysis verified that the ABCG2 expression in tumors of patients with severe depression was 15.6-fold higher than that in tumors from patients with mild depression (Figure 5E).

## DISCUSSION

As multidisciplinary research broadens, investigators have come to realize that cancer is a systemic disease and that the body's mental state, nervous, immune and endocrine systems have an important impact on the occurrence, development and prognosis of cancer [13–15]. Patients diagnosed with cancer are at an increased risk for several common mental disorders as early as the year when diagnosis [16]. Psycho-social distress such as depression, which is a negative form of stress, has also been shown to be associated with poor survival of cancer patients [17, 18]. Thus, new prognostic monitoring strategies with high efficiency are urgently needed.

Recently, the significance of the relationship between depression and cancer has been seriously considered [19]. G. Buccheri indicated that a relationship does exist between depression and patient prognosis, as the survival of depressed patients was significantly lower [20]. Kitagawa R suggested that depression increases the length of hospitalization for patients with malignancies who undergo thoracic surgery [3]. In this study, we showed that depression significantly reduces patient tolerance to chemotherapy. For example, depression significantly increases chemotherapy-induced vomiting and leukopenia and leads to chemotherapy resistant in these patients. This phenomenon reduces the likelihood that patients will follow the advice of their physicians, which would lessen the effects of chemotherapy. We further found that depression is significantly correlated with the PFS of patients with SCLC and that the overall survival time is significantly shortened as the severity of depression increases. Our results suggested that depression should not be considered a mental illness in cancer alone and that its effects make it a novel biomarker for prognosis.

BDNF is a well-studied growth factor that serves many critical functions within the CNS, and serum BDNF is a true response to brain function. As BDNF is expressed in the prefrontal cortex, amygdala and hippocampus [21], and is crucial for neurogenesis and neuronal survival [22]. Erickson has shown a positive relationship between serum protein levels, hippocampal volume and memory performance [23]. In addition, BDNF plays a role in processes such as neuronal maturation, synapse formation and synaptic plasticity, among others, in the brain [24], and has also been implicated in a number of psychiatric disorders such as depression [25]. BDNF has been linked to the mechanism of action of antidepressants [26–28]. Epidemiological evidence and basic research have indicated that stress can affect cancer at multiple levels (e.g., initiation, tumor growth), although the mechanisms of these effects are poorly understood. Previous studies by Sara Capoccia indicated that stress due to social isolation plays a pivotal role in the promotion of breast cancer *in vivo* [29]. Cao L suggested that BDNF appears to be the most selectively responsive to a lack of depression and could therefore serve as a potential mediator of tumor resistance [14]. Therefore, we hypothesize that depression might trigger a mechanistic role for BDNF in cancer progression. Here, we observed that BDNF levels were significantly decreased in patients with a higher depressive state, and with an increase in BDNF, the survival time of patients was significantly prolonged (Figure 2A and 2B). These data are consistent with reports that show that acute immobilization stress induces a rapid increase in BDNF mRNA expression in the hypothalamus, which precedes the activation of corticotropin-releasing hormone (CRH) neurons; this suggests that BDNF is involved early in the regulation of the hypothalamus-pituitary-adrenal (HPA) axis [30]. Through the above results, we confirmed that BDNF is associated with depression and that it affects the survival time of patients.

Chemoresistance dictates the length of survival time, and 75% of SCLC patients will relapse within 6 months due to chemotherapy-resistant [31]. BDNF and its TrkB receptor are known to protect tumor cells from chemotherapy-induced cell death. Thomaz A suggested that BDNF is associated with increased proliferative capabilities, invasiveness, and chemoresistance in several types of cancer [32]. Gao Y showed that BDNF could induce a higher expression of HIF-1α via the activation of TrkB in human Y-79 retinoblastoma cells, which consequently contributed to its effect against chemotherapeutic agent-induced cytotoxicity and apoptosis [33]. However, our results differ from those of the above study in SCLC. Different concentrations of soluble BDNF were added to the culture medium of NCI-H69 cells, and we found that BDNF itself had no effect on cell proliferation, but the chemosensitivity of cells to Cisplatinum improved significantly. In this regard, we believe that the sensitivity of lung cancer cells to Cisplatinum differs from that of nervous system tumors. Additionally, different chemotherapeutic drugs induce distinct death pathways, and growth factors utilize different signal transduction pathways to modulate the effects of chemotherapy on cells.

ABCG2, which is one of the most important ABC efflux transporters, protects normal tissues from xenobiotics via their active expulsion from cells [34]. ABCG2 is ubiquitously expressed in different tissues and contributes to the disposition of a wide variety of endogenous substances and drugs [35]. This protein is also widely known as a transporter that is involved in multidrug resistance. Our results suggested that the upregulation of ABCG2 is likely one of the mechanisms that underlies BDNF-induced tumor resistance. On the mechanism, we believe that BDNF itself does not affect tumor growth, but can reduce hypoxia of tumor, thereby reducing the ABCG2 expression[36]. However, whether other pathways mediate this effect remains to be verified.

In conclusion, we provided evidence to support that the presence of complex psychiatric disorders such as depression will reduce patient tolerance and chemosensitivity in patients with SCLC.

## MATERIALS AND METHODS

### Patients and procedures

In all, 196 patients (125 patients from Henan Cancer Hospital, 71 patients from The First Affiliated Hospital of Soochow University, observed between January 2010 and October 2013) with histologically or cytologically confirmed inoperable non-metastatic [stage IIIB or IV according to the American Joint Committee on Cancer (AJCC) staging system, 6th edition] small cell lung cancer, were considered for this study. During the study, 10 items of invalid data were excluded. Finally, 186 patients were successfully enrolled in the study. Before written consent was obtained, each participant was fully informed as to the purpose of the study. Inclusion criteria were as follows: i) age greater than 18 years; ii) willingness to participate in and complete the tests; iii) awareness of their cancer diagnosis; iv) treatment with initial chemotherapy [typically 6 cycles of a platinum agent: Cisplatin (CP) or Carboplatin (CB)]; v) ability to speak Chinese; vi) no active concomitant cancer or other medical condition; vii) physical ability to cope with the completion of the questionnaires. The demographic information of the patients was collected when they agreed to participate in the clinical research, and clinical examination data were collected both prior to chemotherapy and after chemotherapy (Table 1).

The study design was prospective, descriptive and correlational. All patients completed the General Information Questionnaire to provide general information as well as the Self-Rating Depression Scale (SDS) and the QoL Scale to assess depression symptoms. The study protocol was approved by the Joint Clinical Research Ethics Committee of the Affiliated Cancer Hospital of Zhengzhou University (Zhengzhou, P.R. China). Informed consent was obtained from all subjects prior to the start of the study.

### SDS & QoL assessment

SDS was used to rate the depressive mood of the patients [37]. This questionnaire consists of 20 items selected by factor analysis. It has been translated into a wide variety of languages and its validity and reliability across cultures have been thoroughly assessed. Respondents described how frequently they experienced each symptom on a 4-point scale, as follows: ‘little of the time’, ‘some of the time’, ‘good part of the time’, or ‘most of the time’. The frequency was converted to an integer between 1 and 4, and the total SDS score is the sum of the numbers obtained in response to the 20 questions. All questionnaires were written in Chinese and were completed by the patients themselves. Patients with a score less than 39 on the SDS were designated as normal, patients with a score of 40 or greater were considered mildly depressed, and patients with a score of 50 or more were considered moderately depressed.

We measured the self-reported QoLS with the Functional Assessment of Cancer Therapy-General (FACT-G) [38]. FACT-G contains 28 items with subscales that assess well-being in the previous week across 4 domains (physical, functional, emotional, and social). Scores in the FACT-G range from 0 to 112, and higher scores indicate a better QoL. Patients were each given four different QoL questionnaires and were instructed how to complete them the first day after each chemotherapy cycle. We collected and reviewed the cards, and when reports were found to be incomplete or not sufficiently reliable, we offered assistance to patients so that the quality of the reports could be improved.

### Real-time quantitative PCR

Total RNA was extracted and subjected to reverse transcription using a FastQuant RT kit (with DNase) (Tiangen Biotech, Beijing, China). Quantitative real-time PCR was performed with FastStart Universal SYBR Green Master Mix (Roche Diagnostics, Mannheim, Germany) in a StepOne Plus real-time PCR system (Applied Biosystems, Foster City, CA, USA). The relative expression levels of target genes were calculated after normalization against GAPDH. The primer sequences used for quantitative PCR are listed in Table 3.

**Table 3:** Sequence of primers for mRNA quantification

Gene name	Sequence of primers	Reference sequence (amplicon size)
GST-p1	Forward: 5′-TCCAATACCATCCTGCGTCAC-3′	NM_000852.3 (178 bp)
	Reverse: 5′-CGGGCAGTGCCTTCACATA-3′	
GSTm1	Forward: 5′-GGACGCCTTCCCAAATCTGA-3′	NM_000561.3 (132 bp)
	Reverse: 5′-TACTTGTTGCCCCAGACAGC-3′	
ABCG2	Forward: 5′-TTTCCAAGCGTTCATTCAAAAA-3′	NM_004827.2 (73 bp)
	Reverse: 5′-TACGACTGTGACAATGATCTGAGC-3′	
PPARa	Forward: 5′-GCCTCCTTCGGCGTTCG-3′	NM_001001928.2(146 bp)
	Reverse: 5′-GCTCCAAGCTACTGTGGTGA-3′	
DPYD	Forward: 5′-GGACAGAGTCCAGCTACTGTG-3′	NM_000110.3 (109 bp)
	Reverse: 5′-TGCGCTGTTCCAGATAAGGT-3′	
ABCB1	Forward: 5′-CCCATCATTGCAATAGCAGG-3′	NM_000927.4 (156 bp)
	Reverse: 5′-GTTCAAACTTCTGCTCCTGA-3′	
ABCC1	Forward: 5′-TCTGGTCAGCCCAACTCTCT-3′	NM_004996.3 (123 bp)
	Reverse: 5′-ACTAGGGCTACCAGCCAGAA-3′	
ABCC2	Forward: 5′-TCTGGTCAGCCCAACTCTCT-3′	NM_000392.4 (148 bp)
	Reverse: 5′-ACTAGGGCTACCAGCCAGAA-3′	
ABCC9	Forward: 5′-ACGTATGCTGGAACTGACGG-3′	NM_005691.3 (114 bp)
	Reverse: 5′-GCAGTGAGGACAATGCAAGC-3′	

### Cell culture and BDNF treatment

The NCI-H69 tumor cell line was kindly provided by Dr. Hong Tu (Shanghai Cancer Institute, Shanghai, P. R. China) and was maintained in complete medium, which consisted of RPMI 1640 medium (Hyclone, Logan, UT) supplemented with 10% fetal bovine serum (Invitrogen, Carlsbad, CA, USA), 2 mM glutamine, 100 μg/ml streptomycin and 100 U/ml penicillin. Cells were cultured in a humidified incubator at 37°C with 5% CO_2_.

In order to investigate the effect of BDNF on the growth of the NCI-H69 cell line, the cells were incubated with 1640 medium supplemented with different concentrations of water-soluble BDNF (Sigma Aldrich, Shanghai, P. R. China) at 37°C for 48 h before the experiments were performed. Then, the Cisplatinum sensitivity test was implemented as described below.

### Chemosensitivity test

To assess the chemosensitivity of tumor cells, cell viability was measured by an MTT assay (Colorimetric CellTiter96^®^ AQueous One Solution Cell Proliferation Assay, Promega, Beijing, P. R. China). Cell suspensions were cultured in 96-well flat-bottom microtiter plates at a concentration of 1×10^4^ cells/per well and incubated overnight. Cisplatinum treatments were performed as follows: 10^−8^ M, 10^−7^ M, 10^−6^ M, 10^−5^ M, 10^−4^ M, or 10^−3^ M. Each drug was tested in triplicate, and NCI-H69 tumor cells cultured in complete medium alone were used as blank controls. The cells were incubated for 48 h before the addition of MTT solution (1 mg/ml per well), and OD values were read at 490 nm using a spectrophotometric microplate reader (Bio-Rad, CA, USA). The percent cell viability after treatment with different drug concentrations was calculated as the inhibition rate (mean absorbance of treated wells/mean absorbance of control wells) × 100%. The IC_50_ was calculated by GraphPad Prism v5.0 (GraphPad Software, Inc; P. R. China).

### Western blot

Cell or tumor lysates were obtained and equal amounts of protein lysates from each sample were diluted with loading buffer, denatured, and separated by 10% sodium dodecyl sulfate-polyacrylamide gel electrophoresis (SDS-PAGE) followed by protein transfer to polyvinylidene fluoride membranes (PVDF).

After incubation in a blocking solution (5% nonfat milk powder) in TBST buffer (10 mM Tris-HCl, pH8.0, 150 mM NaCl, and 0.1% Tween20) for 1 h at room temperature, the membranes were immunoblotted overnight with primary monoclonal antibodies (Abs) against either ABCG2 (Santa Cruz Biotechnology Inc., Dallas, TX) or Actin at a 1:1000 dilution at 4°C. The membranes were then incubated for 2 h at room temperature with the appropriate secondary antibody (1:1000 dilution). The protein antibody complex was detected by an enhanced chemiluminescence detection system. The protein expression was quantified by ImageJ software (http:/rsbweb.nih.gov/ij/).

### Immunohistochemistry

For immunohistochemistry, 2-μm-thick sections were de-paraffinized in xylene and rehydrated in graded alcohol solutions as follows: 2×100%, 95%, 75%, 50% and 30% ethanol. After 3 washes in _dd_H_2_O, the sections were placed in 1× antigen retrieval solution (10 mM citrate buffer, pH 6.0) and boiled at 100°C for 10 min. Next, the sections were allowed to cool to room temperature at which point they were treated with 3% H_2_O to block endogenous peroxidase. The sections were then incubated with the primary Abs at 4°C overnight. Primary antibody staining was visualized using the ImPress Universal kit (Vector Laboratories, Burlingame, CA) with NovaRed (Vector Laboratories) as a substrate. The sections were then counterstained with hematoxylin, dehydrated, and mounted. To quantify immunohistochemical staining, ImageJ software (http:/rsbweb.nih.gov/ij/) was used to determine the percentage of tissues that showed immunoreactivity for ABCG2 in microscopy images and in acquired JPEG images (20× magnification with 10 random fields per subject).

### Statistical analysis

Statistical calculations were performed using IBM SPSS statistics 17.0 (SPSS, Inc, Chicago, IL, USA). Descriptive analyses were presented as the means and standard deviations for normally distributed variables. PFS was defined as the duration between the first chemotherapy dose and the date of disease progression or death, while OS was defined as the duration between the first chemotherapy dose and death, loss to follow-up or the current date. Correlation analysis was performed calculating Pearson's coefficient. PFS and OS were calculated by the Kaplan-Meier method and a log-rank test was used to compare different categories. The significance of the differences between the mean values was determined by the *t-test*. The difference in the distribution of ordinal variables was evaluated with the χ^2^ test. A *P-value* < 0.05 was considered statistically significant.
